# Development of In Vitro Bioengineered Vascular Grafts for Microsurgery and Vascular Surgery Applications

**DOI:** 10.1097/GOX.0000000000002264

**Published:** 2019-05-23

**Authors:** Gurtej Singh, John Cordero, Brody Wiles, Miltiadis N. Tembelis, Kai-Li Liang, Miriam Rafailovich, Marcia Simon, Sami U. Khan, Duc T. Bui, Alexander B. Dagum

**Affiliations:** From the * Division of Plastic and Reconstructive Surgery, Department of Surgery, Stony Brook Medicine, Stony Brook, N.Y.; † Division of Plastic and Reconstructive Surgery, Department of Surgery, Stony Brook University School of Medicine, Stony Brook, N.Y.; ‡ Department of Materials Science and Chemical Engineering, Stony Brook University Medical Center, Stony Brook, N.Y.; § Department of Oral Biology and Pathology, Stony Brook University Medical Center, Stony Brook, N.Y.; ¶ Division of Plastic Surgery, Department of Surgery, Stony Brook University Medical Center, Stony Brook, N.Y.

## Abstract

**Introduction:**

The use of vascular grafts is continuing to rise due to the increasing prevalence of coronary artery bypass grafting and microvascular flap-based tissue reconstructions. The current options of using native vessels (saphenous vein) or the synthetic grafts (Dacron) have been unable to manage current needs. In this study, we employed an original tissue engineering approach to develop a multi-layered vascular graft that has the potential to address some of the limitations of the existing grafts.

**Materials and Methods:**

Biomaterials, gelatin and fibrin, were used to develop a two-layered vascular graft. The graft was seeded with endothelial cells and imaged using confocal microscopy. The graft’s architecture and its mechanical properties were also characterized using histology, Scanning Electron Microscopy and rheological studies.

**Results:**

Our methodology resulted in the development of a vascular graft with precise spatial localization of the two layers. The endothelial cells fully covered the lumen of the developed vascular graft, thus providing a non-thrombogenic surface. The elastic modulus of the biomaterials employed in this graft was found to be 5.186 KPa, paralleling that of internal mammary artery. The burst pressure of this graft was also measured and was found close to that of the saphenous vein (~2000 mm Hg).

**Conclusions:**

We were successfully able to employ a unique method to synthesize a multi-layered vascularized graft having adequate biological and mechanical properties. Studies are ongoing involving implantation of this developed vascular graft in the rat femoral artery and characterization of parameters such as vascular remodeling and patency.

## INTRODUCTION

Biological implants are in constant demand due to their ability to repair soft tissue defects resulting from trauma, congenital disease, and oncologic resection.^1^ Their global market share is estimated at more than $5 billion and is expected to grow substantially over the coming years.^2–4^

Biological implants created via tissue engineering have the potential to provide a nearly unlimited supply of tissue while avoiding morbidity related to donor site harvesting. Using a combination of cells and biomaterials, this approach has resulted in the generation of tissues resembling muscle, bone, and skin. However, the development of vascular grafts has been an ongoing challenge. Vascular grafts are in constant need in surgery whether it is to extend the length of a pedicle of a free flap, span a zone of injury in replantation surgery, or bypass disease segment of artery in cardiovascular or peripheral vascular surgery.^5,6^

Synthetic polymers such as Dacron and polytetrafluoroethylene (PTFE) are popular in large-diameter (>6 mm) applications but inadequate for small-diameter (<6 mm) applications with a high rate of thrombosis.^7,8^ Tissue-engineered vascular grafts (TEVG) represent a potential solution to this problem.^9–11^ Multiple strategies have been developed in the creation of TEVGs since their inception in 1986, which include (1) scaffold methods, in which a synthetic material (such as Dacron) is seeded with recipient cells to simulate the tunica intima, (2) decellularized matrices, in which a tissue (typically a blood vessel) is harvested from an animal, chemically decellularized to reduce antigenicity, and then seeded with recipient cells, and (3) self-assembled grafts, in which cell-laden biomaterials are shaped into vessels via methods such as direct molding, folding cellular sheets, and 3D printing.^10^

Despite differences in methods of TEVG formation, no one approach has shown to be most effective. Each strategy has encountered individual difficulties which have prevented their widespread implementation, including lackluster mechanical properties, vessel size limitations, and thrombogenicity. Success of TEVGs has been largely dependent on graft size; medium-sized (12–24 mm diameter) TEVGs have shown early promising results.^12^ On the other hand, small-diameter (<6 mm) TEVGs have not. A clinical trial conducted in the United States and Poland implanted small-diameter TEVGs as vascular access for hemodialysis in 60 patients with failing arteriovenous fistulas. Only 15% retained primary patency at 2 years.^13,14^

Synthesizing small diameter TEVG with satisfactory mechanical properties and long-term patency rates has proved difficult, hindering the integration of TEVGs into clinical applications in reconstructive microsurgery, replantation, coronary artery bypass and grafting, and peripheral vascular surgery.^15^ In this study, we hypothesized and showed that a multilayered approach in conjunction with a dual–half-lumen methodology could be used to construct a small-diameter TEVG. This new methodology allows for the seeding of endothelial cells with standard pipetting technique under direct visualization and allows confirmation of endothelial monolayer, an essential factor in maintaining patency, before graft assembly, without a sacrifice in mechanical strength. We used various assessment methods such as histology, scanning electron microscopy (SEM), elastic modulus, and burst pressure to report on our technique and results.

## MATERIALS AND METHODS

### Cell Culture

Human umbilical vein endothelial cells were cultured at 37^°^C in 5% CO_2_ in EGM-2 BulletKit CC-3162 (Lonza Walkersville Inc., MD). The cells were expanded in T-75 flasks between 5 and 7 passages; the culture media were changed every other day. Before seeding onto vascular grafts, cells were harvested using 0.25% trypsin, and 150,000 cells were added per graft and incubated in static culture for 4 days. In preliminary experiments, several different densities of cells were tested. The current density was found to be optimum in terms of favorable viability of cells and incubation period.

### Construction of Vascular Graft

**Fig. 1.**
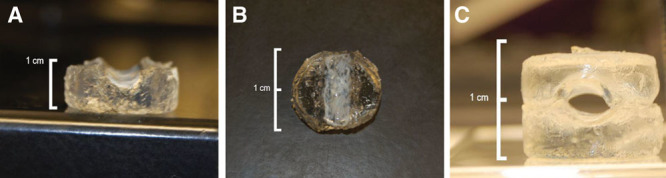
Hemisections of the graft. Use of 2 layers to mimic tunica intima and tunica media layers of the blood vessels with fibrin (top layer) and gelatin (bottom base), respectively. A, Side view of the hemisection. B, Top view of the hemisection. C, Graft generated through fusing 2 hemisections, shown by its axial section.

Several different concentrations of biomaterials including collagen, gelatin, and fibrin were tried in our preliminary experiments. Based on these experiments, gelatin and fibrin were found to have optimum biological and mechanical properties. Gelatin powder from porcine skin (Sigma Aldrich, MO) was utilized to form the outer layer of the vascular graft. It was dissolved in distilled water (10% wt/vol), followed by filter sterilization using a 0.22-micron filter. It was then cross-linked through the addition of microbial transglutaminase (MTG) enzyme (10% wt/vol). Human Fibrin generated from EVICEL Fibrin Sealant (Ethicon Inc., NJ) was used to form the inner layer (lumen) of the graft. Fibrinogen and thrombin were dissolved in equal volumes to produce fibrin. To develop a vascular graft, 3 mL of gelatin/MTG mixture was incubated overnight in a 12-well plate with a 6-mm diameter tube inserted at a depth that results in the development of half lumen of 3 mm in radius. The tube was then removed and any remaining MTG in the solidified gelatin was denatured by keeping the plate at 60^°^C in an oven for 10 minutes. Following that, 75 µL of fibrinogen (1 of the 2 components of fibrin sealant) was uniformly deposited onto this half-lumen, followed by addition of 75 µL of thrombin. The plate was then allowed to incubate for 5 minutes. The cells were then seeded directly on the top of this half-lumen of the graft using a 1-mL pipette. The cells were incubated for a period of 2 hours. Similarly, another half-lumen was generated. The 2 half-lumens were then joined together through the addition of fibrin sealant (thus creating a 2-cm-long and 6-mm-diameter vascular graft). This was followed by further incubation for a period of 4 days (Fig. 1).

### Histology and SEM Imaging

The samples were fixed in 10% formalin, followed by paraffin embedding and sectioned into 5-µm-thick slices. These slices were stained with hematoxylin and eosin and Masson’s trichrome. Furthermore, gelatin fibrin ultrastructure was characterized using SEM. Briefly, 5-µm sections upon being mounted on glass slides were stained with 1% Os(OH)_4_, followed by dehydration processes with ethanol. The slides were then sputter coated with Au/Pd particles and were imaged using Crossbeam 340, Carl Zeiss Microscope.

### Confocal Microscopy Imaging

The endothelial cells lining the lumen were labeled with Hoechst 33342 dye and Alexa Fluor 488 phalloidin stain for 1 hour. Following washing with saline solution, the cells were imaged using Upright Confocal Microscope, Leica TCS SP8 X.

### Mechanical Testing and Burst Pressure Measurements

**Fig. 2.**
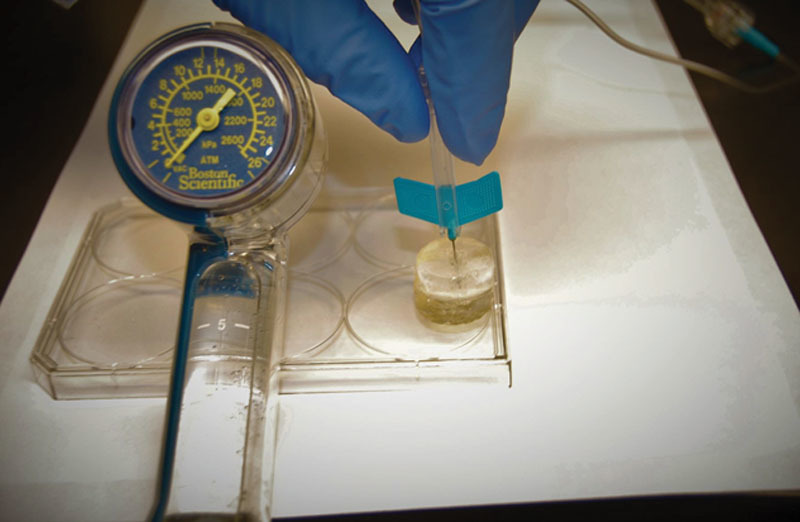
Burst pressure measurement of the vascular graft using angioplasty balloon inflation device.

Several rheology studies were conducted to determine the elastic modulus and shear stress of both the biomaterials involved—gelatin (after being cross-linked) and fibrin. The burst pressure was measured using an angioplasty balloon inflation device (Fig. 2). Both ends of the graft were sealed. Then saline was inserted from the top, and the pressure at which the graft fell apart was recorded.

### Image Acquisition and Analysis

The images of all histological stains at different magnifications (4×, 10×, and 40×) were recorded using a Nikon Eclipse E800 microscope.

## RESULTS

### Histology and SEM Imaging

**Fig. 3.**
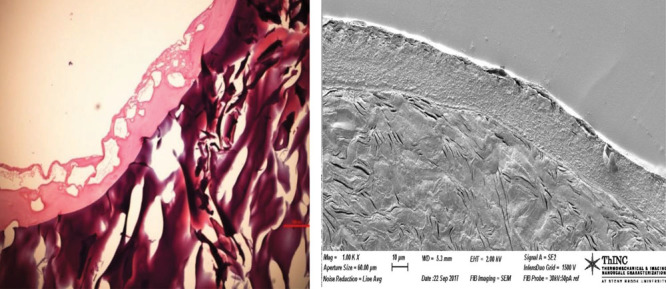
Trichrome-stained and SEM images of the gelatin–fibrin interface of the half lumen. The top layer in both these images corresponds to fibrin, whereas the bottom layer corresponds to gelatin.

Distinct gelatin–fibrin interfaces were observed in both imaging modalities (Fig. 3).

### Biological Characterization of the Graft

**Fig. 4.**
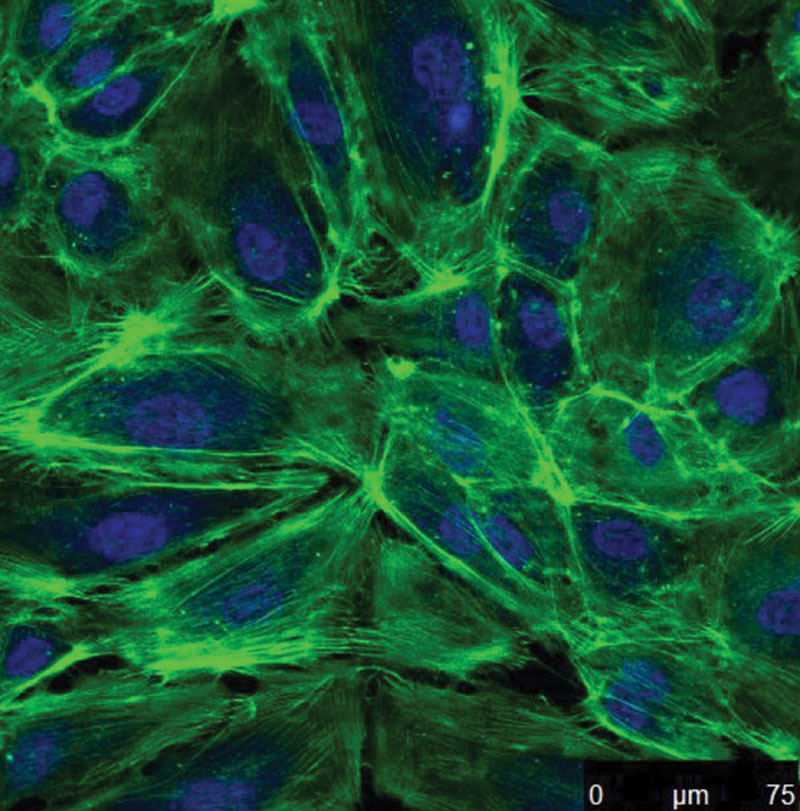
Developed vascular grafts were stained for Hoechst 33342 (nuclei dye—blue) and Alexa Fluor 488 phalloidin (stain for actin filaments—green). Image was acquired at 40× magnification using an upright confocal microscope.

Following 4 days of incubation of cells onto the fibrin-composed lumen, a confluent endothelium was obtained as demonstrated in Figure 4. A well-defined network of actin cytoskeleton connections to the underlying fibrin matrix provide a suitable environment for this graft to undergo vascular remodeling and maturation.

### Mechanical Characterization of the Biomaterials Employed

**Fig. 5.**
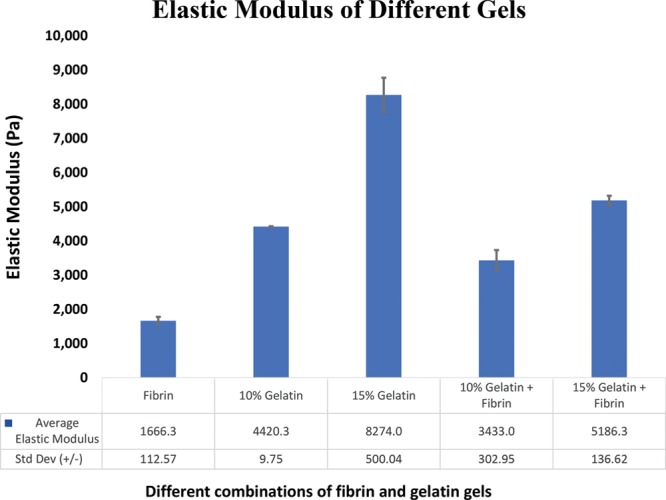
Comparison of elastic modulus of various gels employed in our study.

The gelatin–fibrin combination used in the final design (10% gelatin base with overlaid fibrin coating) had an elastic modulus of 3433 pascals (Pa) (Fig. 5). Increasing the composition of gelatin base from 10% to 15% while maintaining the fibrin layer resulted in an approximately 1.5-fold increase in elastic modulus to 5186.3 Pa. Gelatin bases constructed without the use of overlaid fibrin layer possessed higher elastic moduli relative to their multilayered counterparts (10% gelatin base, 4420.3 Pa; 15% gelatin base, 8274 Pa). The elastic modulus of the isolated fibrin layer used in graft construction was 1666.3 Pa.

### Burst Pressure Measurements

Constructed grafts were noted to burst at internal pressures of approximately 2000 mm Hg (n = 3). Bursting in all trials originated within the plane of the fibrin sealant between the 2 half-lumens; no leak was visualized through the gelatin of either half lumen at the time of graft bursting.

## DISCUSSION

TEVGs have found success in medium-to-large-diameter applications; however, their results in small-diameter applications have been discouraging. This has spurred motivation to engineer a graft that exceeds the patency and mechanical properties of native vascular conduits, allows for unlimited supply of vascular grafts, and obviates the morbidity associated with their harvesting. In this study, we describe the biological and mechanical properties of a small-diameter TEVG constructed using unique a dual–half-lumen methodology.

The endothelial cells used in this study successfully grew along the curved surface of this 2-cm-long and 6-mm-diameter graft, and there was no neointimal hyperplasia observed (which is usually associated with smooth muscle cells). These cells (human umbilical vein endothelial cells) were chosen for their easy availability and quick proliferation in in vitro studies. However, our methodology could be employed for the lining of both venous and arterial cells onto vascular grafts and for any length or diameter of graft. In future clinical applications, we foresee the use of the patient’s native progenitor cells from the blood or marrow used for seeding these custom-designed grafts.

A crucial factor in maintaining patency is the attainment of a nonthrombogenic endothelial cell monolayer, as it mitigates against adherence of blood proteins and activation of the coagulation cascade and regulates vascular tone. Furthermore, it is generally accepted that a stable endothelium must be present before implantation.^16^ In this study, a confluent antithrombogenic endothelial monolayer was achieved as demonstrated via confocal microscopy following 4 days of incubation (Fig. 4). This layer was limited to the top fibrin layer. No penetration of endothelial cells into the deeper gelatin layer was observed as demonstrated by histological and SEM imaging, showing the maintenance of structural integrity of the 2 layers (fibrin and gelatin).

Several mechanical properties key to successful in vivo implementation were investigated after the construction of our TEVG. Historically, elasticity has been an important marker of graft stability. A significant difference in elasticity between an implanted native vessel and an implanted conduit results in turbulence at the sites of anastomosis. This ultimately precipitates graft failure secondary to complications such as graft ectasia, aneurysm, and thrombus formation.^17^ Elasticity confers resistance to high burst pressures and compliance, which is essential under conditions of high pulse pressures.^18^ Adequate elasticity is also required to convert the pulsatile flow generated by the heart into a continuous stream by storing potential energy during systole and applying it to the circulation during diastole.^19,20^ The elasticity of our TEVG was characterized using elastic modulus, a measure of resistance to deformation when a stress is applied. More simply, it may be defined as the ratio of tensile strength to tensile strain. Our multilayered vascular construct composed of fibrin and gelatin achieved a modulus as high as 3.433 kPa, which is about half of the value previously measured in the internal mammary artery (IMA); 6.9 ± 1.7 kPa/unit strain when subjected to 100 cm Hg.^21^ However, by altering our biomaterial concentrations, we achieved a modulus of 5.186 kPa, paralleling the elasticity of the IMA. These data are encouraging as it suggests that our construct may achieve physiologic elasticity simply by altering the concentrations of graft components.

Adequate burst pressure is another measure of graft stability that is critical in the development of TEVGs. Inadequate wall strength proposes a dangerous scenario where a graft is unable to withstand arterial pressures and leaks or becomes aneurysmal and ruptures after being implanted. Native vascular grafts demonstrate burst pressures ranging from 1600 to 2400 mm Hg for great saphenous vein (GSV) grafts and 3200–4225 mm Hg for IMA grafts.^22–25^ These considerable ranges in native vessel mechanical properties have been attributed to the effects of age-related changes, atherosclerosis, and inherent differences in vessel diameter.^26,27^ Previous models of TEVGs have had no problem achieving adequate burst pressure. Most constructs have shown the ability to withstand pressures >1500 mmHg^17,28^ with others demonstrating burst pressures upward of 3500.^22,24^ Thus, it is to be expected that a new TEVG such as the one created in this experiment would have burst pressures in the 1500–3500 mm Hg range. Our graft’s burst pressure of approximately 2000 mm Hg is most represented by the strength of the fibrin sealant joining the 2 half-lumens rather than of the actual graft wall. Because bursting occurred between the half-lumens first before occurring through the gelatin wall, we postulate that different types of half-lumen sealant would result in different burst pressures for grafts made using this dual–half-lumen method. It is worth noting that for any given method of TEVG, graft burst pressure is variable and is known to change with differing graft length.^22^ Additionally, according to Laplace’s law, differences in graft radius will also influence wall tension, and thus, burst pressure. These principles are important when attempting to extrapolate a graft’s performance at differing diameters and lengths and suggest that there likely exists the need for several different methodologies to construct successful grafts of varying diameter.

Autologous vessels have historically been notorious for their suboptimal long-term patency rates. The often-used GSV has a patency rate of only 57%–61% after 10 years, compared with the IMA which has 10-year patency rates that approach 90%.^22,29,30^ The striking differences between the viability of the GSV and IMA grafts are typically attributed to increased rates of acute thrombosis, intimal hyperplasia, and accelerated rates of atherosclerosis in vein grafts.^29^ Although strategies such as antiplatelet therapy and cholesterol control have led to improved patency rates, there exists the opportunity for TEVGs to improve on long-term graft patency. Polyglycolic acid scaffold-based TEVGs have demonstrated 4-week patency rates approaching 100% in porcine models,^31^ and polycaprolactone/chitosan-based grafts have shown promising results in sheep.^32^ However, TEVGs have had variable results when implemented in humans, and there is currently no small-diameter TEVG that has shown promising results in human subjects.^12–14^ Commercial products such as Lifeline Vascular Graft, GORE Propaten, and Humacyte Acellular Vessel suffer from several limitations including poor long-term patency, extensive construction times, and the use of xenograft or allogenic cells. Considering, there are around 1.4 million people per year in the United States alone in the need of vascular grafts, the need to explore new ways of synthesizing biocompatible grafts with long-term patencies and short production time is paramount.

## CONCLUSION

**Fig. 6.**
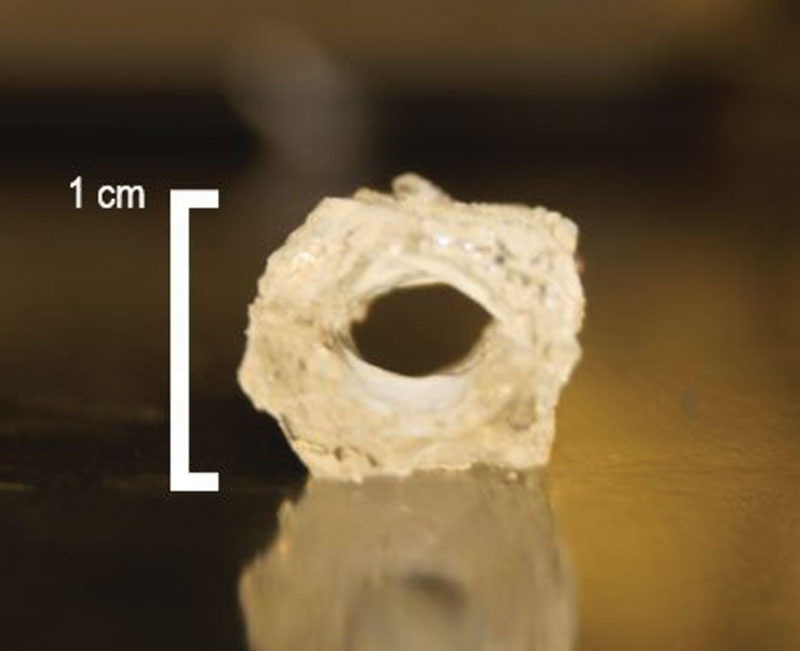
Final vascular graft was obtained by cutting excess thickness on the sides for used in implantation studies in animal’s models.

In this preliminary proof of concept study, we have shown that our new multilayered approach, in conjunction with a dual–half-lumen methodology, can be used to construct a small-diameter TEVG. The development and confirmation of a functional, adherent endothelium before implantation is essential to inhibit thrombosis. The graft’s mechanical properties match favorably to GSV and IMA conduits. It is comparable with regard to elasticity and bursting strength and can function without macroscopic evidence of burst, leak, or structural damage. We are currently in the process of modifying the graft (Fig. 6) to reduce its thickness and to investigate its in vivo viability. We intend to implement this graft in rat femoral arteries, followed by characterization of parameters such as vascular remodeling and patency.

## ACKNOWLEDGMENTS

The authors thank Yan Ji (Research Histology Core Laboratory) and Ya-Chen Chuang (ThINC facility at AERTC) at Stony Brook University with help at histological samples preparation, confocal and SEM images acquisition. The authors are equally grateful to Alice Shih with help in cell culture.
